# Forceps-Assisted Endoscope Rigidity Reinforcement for Difficult Duodenal Intubation due to Cascade Stomach

**DOI:** 10.14309/crj.0000000000001862

**Published:** 2025-10-13

**Authors:** Nobutaka Doba, Kosuke Shibayama, Shinzo Abe, Daiki Sakuma, Masanobu Someya, Kazuto Komatsu, Shin Maeda

**Affiliations:** 1 Department of Gastroenterology, Yokosuka City Hospital, Nagasaka, Yokosuka, Japan; 2 Department of Gastroenterology, Yokohama City University Graduate School of Medicine, Yokohama, Japan

**Keywords:** ERCP, cascade stomach, duodenal intubation, forceps, scope rigidity

## Abstract

A 92-year-old woman underwent endoscopic retrograde cholangiopancreatography (ERCP) for acute cholecystitis and bile duct stones. A cascade stomach caused significant difficulty in duodenal intubation, despite multiple standard maneuvers. As a large-diameter overtube was unavailable, forceps were inserted through the accessory channel to increase endoscope rigidity. This technique minimized scope looping and enabled duodenal access within 2 minutes. Biliary cannulation, sphincterotomy, and drainage were completed successfully. The same method was used in a subsequent ERCP with similar success. This case demonstrates that forceps-assisted rigidity reinforcement is a simple, effective, and equipment-free option for managing duodenal intubation difficulties during ERCP.

## INTRODUCTION

Endoscopic retrograde cholangiopancreatography (ERCP) is a well-established procedure widely used for the diagnosis and treatment of biliary and pancreatic diseases.^1^ Successful ERCP requires duodenal intubation using a side-viewing endoscope. However, anatomical variations such as a cascade stomach or a large hiatal hernia can hinder the advancement of the scope through the stomach, posing a challenge to accessing the major papilla.^2^

A cascade stomach is a morphological abnormality characterized by kinking and sagging of the gastric body, resulting in the pooling of fluid or contrast in the upper stomach followed by stepwise flow.^3^ This configuration often leads to loop formation and disruption of the endoscope's axis, which is particularly problematic for side-viewing endoscopes that have limited maneuverability.

To overcome this difficulty, the use of a large-caliber overtube has been reported. Saito et al described a case in which a colon overtube was used successfully to facilitate duodenal intubation in a patient with a cascade stomach.^4^ However, such specialized equipment may not be available in all institutions.

We report a case in which duodenal intubation during ERCP was extremely difficult due to a cascade stomach, but was ultimately achieved by inserting forceps through the accessory channel to reinforce endoscope rigidity and improve axis control.

## CASE REPORT

**Figure 1.**
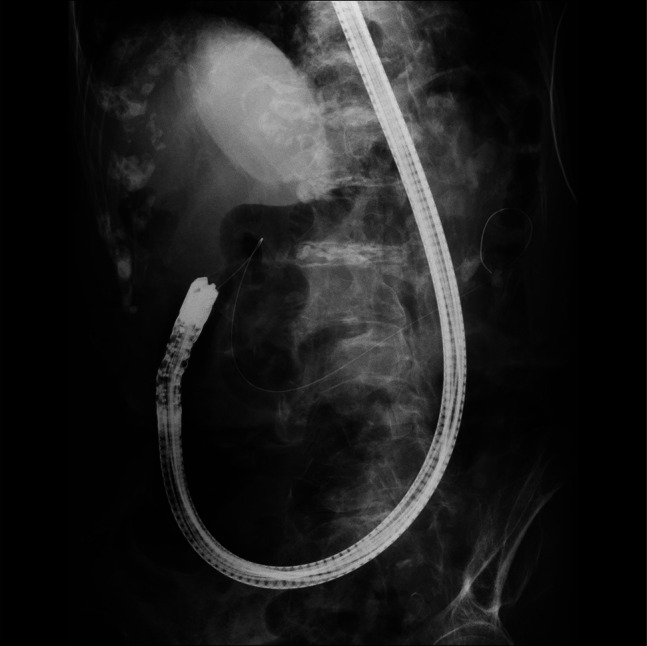
Fluoroscopic image showing marked prolapse of the gastric body into the pelvic cavity, consistent with a cascade stomach.

A 92-year-old woman underwent ERCP for acute cholecystitis and common bile duct stones. At the beginning of the procedure, the gastric body was observed to be markedly prolapsed into the pelvic cavity, consistent with a cascade stomach (Figure 1). Duodenal intubation using a side-viewing endoscope was attempted, but the scope formed a significant loop in the caudal direction. Although the pylorus was barely visible, intubation was extremely difficult.

**Figure 2.**
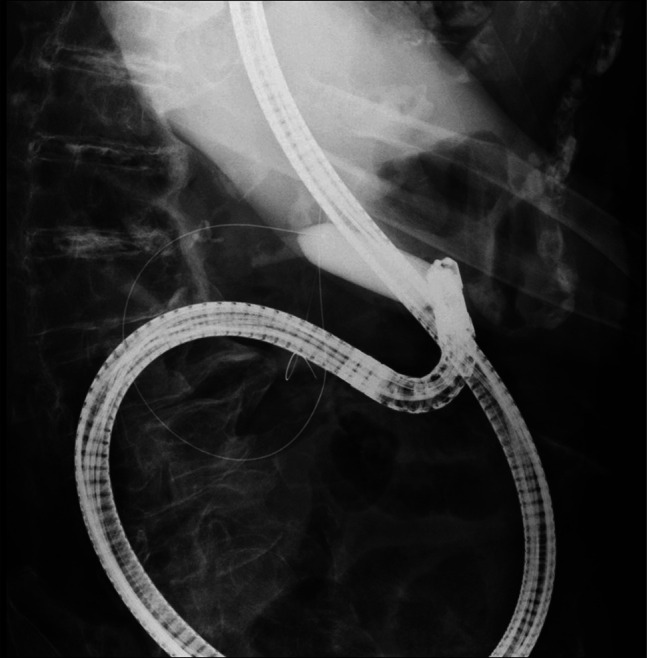
Fluoroscopic image during attempted duodenal intubation using balloon-assisted technique.

Multiple conventional techniques were attempted, including manual abdominal compression, positional change from the prone to the left lateral decubitus position, gastric decompression, exchange of the endoscope, and traction-assisted methods using guidewires, catheters, and balloons. However, despite nearly an hour of efforts, duodenal intubation could not be achieved (Figure 2).

Although previous reports have shown that colon overtubes may be effective for duodenal intubation in cascade stomach cases, our facility did not have such equipment available.^4^

Based on our previous experience in cases where colonoscope insertion was difficult with scopes lacking a variable stiffness mechanism, in which the passage of forceps through the accessory channel increased the rigidity of the endoscope and improved insertion, we applied the same technique in this case.

**Figure 3.**
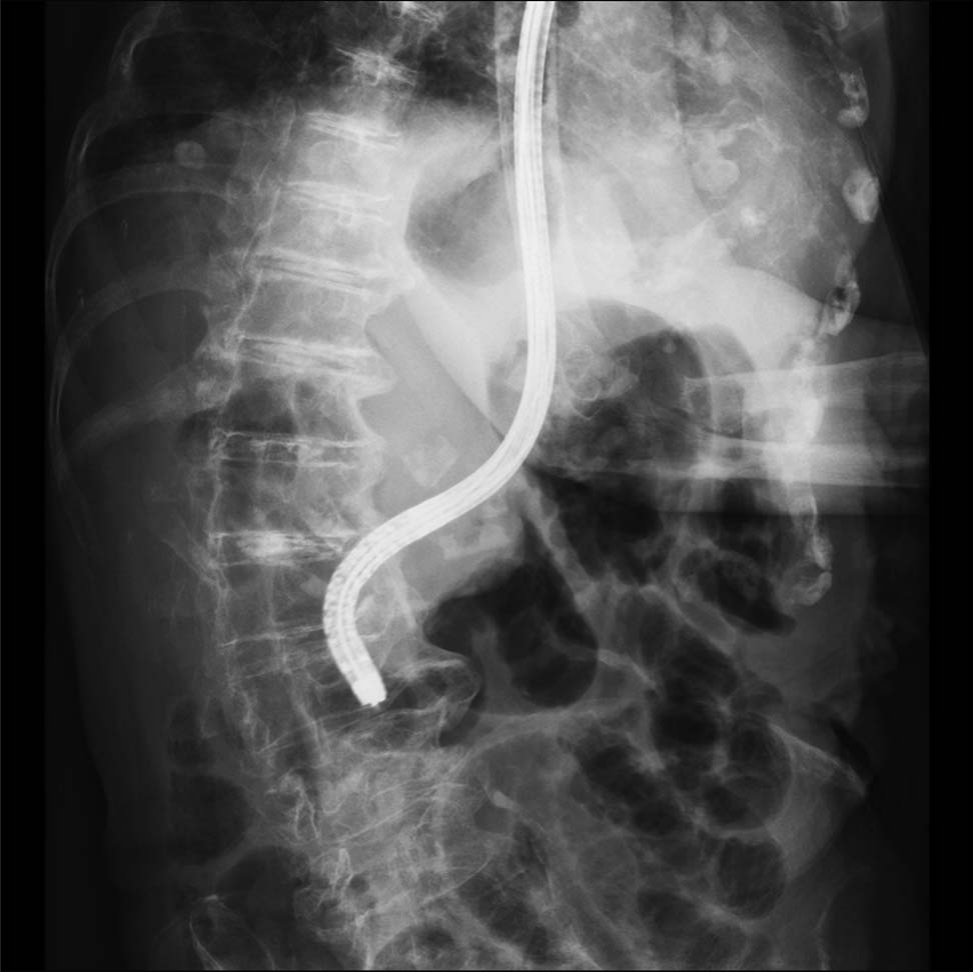
Fluoroscopic image showing reduced scope bending after forceps insertion, enabling successful duodenal intubation.

The insertion of forceps significantly reduced the scope's excessive bending and improved visualization of the pylorus, allowing successful duodenal intubation within approximately 2 minutes (Figure 3). Subsequent biliary cannulation, endoscopic sphincterotomy, and endoscopic biliary drainage were performed without complications.

Endoscopic retrograde gallbladder drainage was attempted but was unsuccessful, and percutaneous transhepatic gallbladder drainage was performed the following day.

One week later, a second ERCP was performed for common bile duct stone removal. From the outset, the forceps-assisted technique was employed, which again allowed successful duodenal intubation within approximately 3 minutes (Video 1). Endoscopic papillary large balloon dilation and stone extraction were completed without difficulty.

Video 1 Difficult duodenal intubation in a cascade stomach was overcome using the forceps-assisted rigidity reinforcement technique.

## DISCUSSION

This case highlights the successful use of temporary endoscope rigidity reinforcement via forceps insertion to overcome duodenal intubation failure during ERCP in a patient with a cascade stomach.

Cascade stomach is characterized by an altered gastric body morphology that predisposes the endoscope to loop formation and axis deviation, thereby complicating scope advancement—particularly with side-viewing endoscopes.^3^ In this case, standard maneuvers—including abdominal compression, patient repositioning, gastric decompression, endoscope exchange, and various traction techniques—were all ineffective in achieving duodenal intubation.

Although the use of overtubes has been reported as an effective strategy for difficult intubation, overtubes may not be routinely available in all institutions.^4^ By contrast, the technique described herein—reinforcing endoscope rigidity by inserting forceps through the accessory channel—can be implemented using standard endoscopic equipment, without the need for specialized devices. Initially adapted from techniques used in difficult colonoscopy cases, this method was successfully applied to a side-viewing endoscope in the context of ERCP.

Notably, previous studies have suggested that excessive flexibility of endoscopes can lead to instability during insertion, and that temporary internal stiffening may improve control.^5^ Our approach aligns with this concept. Side-viewing endoscopes lack variable stiffness functionality, limiting the ability to adjust the rigidity of the scope itself. Therefore, internal stiffening through forceps insertion represents a rational and practical alternative.

In addition to the forceps-assisted technique, several other methods have been reported or are commonly used by experienced endoscopists to overcome duodenal intubation difficulties, particularly in cases involving cascade stomach or significant gastric looping. These techniques include abdominal compression, patient repositioning, scope exchange, guidewire-assisted insertion, balloon-assisted traction, and overtube use.^4^ Particularly in the guidewire-assisted technique, a soft guidewire or catheter is first advanced into the duodenum, and the endoscope is then inserted along the guidewire or catheter. Similarly, balloon-assisted techniques involve positioning an inflated balloon within the duodenum to provide gentle traction and facilitate passage of the scope through the pylorus. A summary of these techniques is presented in Table 1 to guide clinicians facing similar challenges. Table 1 summarizes commonly employed techniques to facilitate duodenal intubation using a side-viewing endoscope, particularly in cases where standard insertion is difficult due to looping or complex anatomy. Each method is compared in terms of mechanism, cost, feasibility for solo operators, and key limitations.

**Table 1.** Comparison of techniques used to facilitate duodenal intubation with a side-viewing endoscope

Technique	Mechanism	Cost	Solo operator feasibility	Limitations/precautions
Abdominal pressure	Manually straightens gastric loops and stabilizes the scope shaft	None	Requires assistant (manual compression)	Requires skilled assistance
Positional change	Alters gastric anatomy and gravity to facilitate insertion	None	Requires assistant (repositioning under sedation)	May be insufficient depending on individual anatomy
Scope change	Changing to a scope with different stiffness or diameter to ease insertion	None (uses existing scopes)	Possible	May require reattempt; effectiveness depends on case and scope selection
Use of guidewire/balloon catheter	Guidewire or balloon catheter is used to guide or anchor the scope	High	Requires assistant (device setup and coordination)	Availability may vary by institution
Overtube use	Prevents loop formation and facilitates reinsertion of the scope	High	Requires assistant (device setup and handling)	Requires reinsertion; may not be routinely stocked at some institutions
Forceps insertion (this case)	Inserting forceps into the channel increases shaft rigidity	Low (uses existing instruments)	Possible	Limits suction and use of other therapeutic accessories

The forceps-assisted method permits scope advancement without the need for excessive force, which may enhance procedural safety. However, this technique has potential limitations, including restricted visualization and decreased maneuverability due to the presence of the forceps within the working channel. These drawbacks should be considered when selecting the most appropriate strategy.

In conclusion, forceps-assisted endoscope rigidity reinforcement is a simple, effective, and device-independent method to facilitate duodenal intubation during ERCP in patients with cascade stomach. This technique may be particularly useful in settings where overtube use is impractical or unavailable.^4^ Further studies are warranted to validate its clinical utility, safety profile, and broader applicability.

## DISCLOSURES

Author contributions: N. Doba: Conceptualization, writing—original draft preparation, writing—review and editing, visualization; K. Shibayama: Data curation; S. Abe, D. Sakuma: Investigation; M. Someya: Data curation; K. Komatsu and S. Maeda: Supervision. N. Doba is the article guarantor.

Financial disclosure: None to report.

Informed consent was obtained for this case report.

## ABBREVIATION:

ERCP: Endoscopic retrograde cholangiopancreatography
